# Analecta

**Published:** 1842-03-26

**Authors:** 


### ANALECTA.

*Six cases of Diarrhœa treated with Monesia.** By Q. Gibbon, Salem N. J.—*Case 1st.* Mrs. K. Married. Had laboured under chronic diarrhœa accompanied with ulceration of the intestines for two years. Three or four months ago she was put upon small doses of acet. of morphia, which after several weeks continuance arrested the disease, and the patient remained for two months apparently perfectly well. At the expiration of this time the disease suddenly returned, and soon regained all its former activity. The extract of monesia was now commenced in doses of 5 grs. three times a day, and afterwards increased to 7 grs. four times per day, and continued for ten days, but without any other benefit than a slight decrease of the discharge for the first two days. The medicine was then discontinued, and the morphia substituted with its former success.

*Case 2d.* A child of W. H., affected with diarrhœa of 18 months standing, had used anodynes, alteratives and astringents freely, but without permanent benefit. The ext. monesiæ given in doses of two grs., three times per day, assisted by the warm bath and frictions with flannel, checked the discharges in two days, and removed all traces of the disease in two weeks.

*Case 3d.* J. M., aged 70—health delicate, had a diarrhœa, with short intermissions, for six months—had been treated with creta cum hydrarg. with but temporary relief. Took six grs. of monesia four times per day, from the 2d to the 10th of November, assisted by a few grs. of pulv. doveri.,

*A detailed notice of the monesia by Dr. Martin St. Ange, will be found at p. 207 of vol. 3d of this journal. For reports of cases treated with monesia, see also pp. 215, 441, 517, 573, of the same volume.

occasionally, at bed time. The discharge ceased in two days, and upon the tenth he expressed himself perfectly well.

*Case 4th.* Mrs. J. Diarrhœa of four or five days continuance—slight fever, tormina, stools bloody and slimy. Had resorted to no previous treatment. Took 8 grs. of ext. mones. three times the first day, which checked the discharge, and allayed all unpleasant symptoms.

*Case 5th.* A child of four years of age, affected with bowel complaint which had commenced in the summer, and continued unchecked until November, though treated with a variety of astringents, and subjected to counter-irritation upon the abdomen. The monesia was given in doses of 2 grs. five times daily for six days, with the effect of gradually diminishing the discharges, changing their colour and consistence, and creating a vigorous appetite, which the child had not previously enjoyed. Having no more of the remedy, I was compelled to relinquish it. Dover's powder and flannel frictions were substituted, and I had the satisfaction of seeing my patient perfectly relieved in another week.

*Case 6th.* In this case the remedy entirely failed as in the first case. It was one of diarrhœa supervening upon phthisis pulmonalis, and had been previously treated by creta cum opio, acet. of lead., acet. of morphia, and several of the vegetable astringents, without benefit. The monesia was prescribed at first to the amount of 15 grs. and afterwards increased to 40 grs. per day, and persevered in for three weeks—when, as no abatement of the symptoms was perceived, the remedy was discontinued at the request of the patient.

*Remarks.*—That the above cases may be entitled to their just weight in the evidence now accumulating both for and against this new remedy, it may be well to state that the few last doses taken by Mrs. K. in the first case, occasioned slight nausea accompanied by a slight burning in the stomach. With this exception, I have not witnessed any of the irritating effects detailed by St. Ange. My doses, however, were not so large as he prescribed. The above cases lead me to infer that monesia is more astringent than tonic in its operation.—*Am. Med. Intel., Jan.*, 1842.

---

*Case of Complete Paralysis of the Seventh Pair of Nerves.* By Dr. C. James.—A young woman, 22 years of age, was seized with paralysis of the left side of the face, involving all those parts which are supplied by the seventh pair of nerves. The features were distorted and drawn towards the right side. She had no command over the motion of the left side of the face, eye-brow or eyelid. The upper lip of this side hung relaxed, and the lower was equally paralysed in its left half, and hung forward, giving issue to the saliva.

Galvanism was employed for the cure of this affection. One needle was fixed in the parotid gland, and the other was successively placed in the supra-orbitar, infra-orbitar, and mental foramen, and a stream of galvanism from Clark's galvanometer passed through and along the diseased nerves. Instead of relieving, the galvanism appeared to aggravate the affection, for by the sixth sitting, the right side of the face became equally affected with the left, and she gradually, but completely, lost the power of modulating the features, moving the lips, the eyelids, or the eyebrows.

At this stage of the affection her features appeared quite regular, but immovable, and quite incapable of expressing any of the emotions of the mind. The eyelids were wide open, exposing a greater portion of the globe of the eye, and she could not close them. The eyebrows hung relaxed over the eyes. The nostrils were relaxed, and during forcible inspirations were pressed by the air against the septum, so as to act as valves and prevent the access of air to the lungs. The lips hung quite relaxed, and her speech was very indistinct, from the impossibility of articulating the labial letters. At every respiratory movement, the lips were blown out or carried in against the teeth, according to the direction of the current of air. The cheeks were relaxed and pendant. Mastication was very troublesome, for the food got between the relaxed cheeks and the gums, and required to be pushed between the teeth by means of the fingers.

Three days after the paralysis was complete on both sides, by the continuance of the galvanism, symptoms of amendment were visible, the features began to be drawn to the left, or first affected side; and in ten days after, the paralytic affection of that side was almost completely removed. It was not, however, till sixteen days after this that the paralysis was removed entirely from the right side of the face also, and the patient was declared cured. The patient had thirty applications of the galvanism before the cure was effected. A few more sittings were, however, given, in order to prevent a relapse.—*Edinburgh Med. and Surg. Jour., from Gaz. Med. de Paris*, 18th Sept., 1841.

---

*Successful case of Amputation at the Hip-joint.* By M. TEXTOR.—[The following case is illustrative of the fact pointed out by Sir B. Brodie, that the vessels are occluded to a very considerable distance beyond the sphacelus, in cases of advancing mortification, while it proves that—although the recurrence of gangrene in stumps, is, in all probability due to this circumstance—incisions carried through the obliterated vessels are not in every instance unfortunate in their results.]

A man was seized with gangrene of the leg, for which the limb was amputated through the knee-joint. The gangrene, however, seized the stump, and M. Textor was obliged to amputate the thigh at the hip-joint. The only unusual occurrence noticed during the time of the operation, was that the crural artery was plugged up with a fibrinous clot, notwithstanding which, a ligature was passed around it. No unfavourable symptom followed; and four months after the operation, the man had so far recovered his health as to be able to walk about the court of the hospital.—*Ibid., from ibid.*, 4th Sept., 1841.

---

*Two cases of Excision of the Callus, in badly united Fractures.* By Professor PORTAL.—A man, 32 years of age, fell from a cart and fractured his tibia and fibula nearly in their middle. Inflammation, followed by purulent infiltration, attacked the limb, and as the patient was very restless, after the cure was effected it was found that the fragments of the bone had united in an irregular angular manner. Professor Portal first tried to break the callus across, in order to reset the limb, but not succeeding in this, he cut down on the irregular angular projection by a vertical incision, and uncovering the bones, passed a chain saw round them and removed about an inch of the bone. The wound united by the first intention; the limb was kept carefully extended, and in forty-eight days the patient was dismissed cured. The limb was shortened a very little.

The other case was that of a woman, who had received a compound fracture of the upper third of the thigh bone. On account of the violence of the inflammation the limb was at first semiflexed, and when this had abated it was placed in the extended posture. After a period of twenty-eight days the bandage was removed, and it was found that the union had taken place irregularly, the broken ends forming an angle at their union. As it was found impossible to rupture the callus which had been thrown out, the ends of the bone were cut down on, a chain saw passed first around the upper fragment and about an inch and a half removed, and then around the lower, and half an inch of it cut off. The limb was then maintained in a state of permanent extension. The wound suppurated and became covered with an eschar, but no other unfavourable symptoms followed; and fifty-five days after the operation she was dismissed cured, with the limb shortened to the extent of two finger breadths, but perfectly serviceable.—*Ibid.*, *from ibid.*, 18th Sept., 1841.

---

*Incipient Phthisis treated with Emetics.* By C. M. Durrant, M. D.—Philis Strowger, æt. 19, a servant, of sallow complexion and leucophlegmatic habit: has lost a brother from consumption, and she herself has been the subject of a winter-cough for two years. On presenting herself for examination about six weeks since, she stated she was much harassed with a severe cough and shortness of breath, which she attributed to cold: complained also of soreness over the chest, with occasional "darting pains" between the shoulders; violent palpitation of the heart on the slightest exertion, which affects her also when in bed, and especially on first awakening in the morning; extremities generally cold; eyes dull, and surrounded with dark broad areolæ; head not painful but subject to giddiness; tongue clean, pale and indented with the teeth; appetite moderate; no thirst; bowels generally pretty regular; urine natural in quantity, depositing a lateritious sediment on cooling; catamenia appeared at sixteen, since which she had been very irregular, both in reference to quantity and period of recurrence; had considerable leucorrhœal discharge; pulse rapid; action of heart quick, jerking, and audible, over entire chest, otherwise normal; ribs under right clavicle did not appear to move so freely as on the opposite side, where also there existed slight dulness on percussion. I was unable to detect in this instance any flattening of the infraclavicular region. At the same spot the vesicular murmur was decidedly more feeble than under the opposite clavicle, together with a well-marked, prolonged, and roughened state of the expiratory sound; voice and cough shrill and acute, and more resonant than natural, under both clavicles, especially the right; could take a full inspiration without pain or increase of cough; had never had hæmoptysis, and did not expectorate; ankles swelled at night.

She was ordered a mild emetic every morning an hour before breakfast. Mist. Ferri Co. ℥i. ter die; a pill every night of Aloes, Ext. Conii, et Pil. Hydrarg.; and to sponge the chest freely night and morning with the turpentine and acetic acid liniment, as recommended by Drs. Stokes and Hughes.

After continuing the above remedies for a week, I added the following note. Cough greatly relieved; no expectoration; respiration much easier; vesicular murmur under right clavicle less rough, and nearly as loud as on the opposite side; pain in chest greatly benefitted by the liniment; bowels open; catamenia still absent; leucorrhœa diminished: confesses in warm terms the relief afforded by the emetics. Ordered to continue their use on alternate mornings; to take two grains of iodide of iron thrice daily, and a pill every night of Ext. Conii, Aloes, et Ferri Sulph. From this treatment the symptoms rapidly improved; and on discontinuing the treatment at her own request, the chest in every respect was perfectly normal. The catamenia, however, had not returned.—*Medical Gazette, Dec.* 31, 1841.

[Emetics are sometimes a very good, though most disagreeable remedy in early phthisis, but we must watch their effects and discontinue them if the stomach suffer. We have seen much evil from their careless administration in this disorder. They are useless in highly constitutional phthisis, and not fitted for those cases in which there is much bronchitis accompanying or preceding the consumption.]

---

*A Case of Stricture of the Trachea.* By W. C. Wotrhington, Esq., Senior Surgeon to the Lowstoft Infirmary. Communicated by James Copland, M. D., F. R. S.—The patient, an agricultural labourer, aged 49, first came under the notice of the author in August, 1837. Four years previously he had contracted syphilis, for the cure of which mercury had been administered, but to an immoderate extent. During twelve months immediately previous to his putting himself under the author's care he had been confined to the house. The state of his respiration most especially attracted the author's attention, both as regarded the peculiarity of the noise attendant upon inspiration, and the very painful effort required for its accomplishment. The sound closely resembled that produced by an unsound horse, called "a roarer;" suggesting the idea that the air passed through a tube of narrow calibre. Each inspiration occupied ten seconds, and was obviously effected at the expense of very powerful exertion of all the muscles about the larynx. Utterance was hoarse and rough, and a troublesome cough was present. The stethoscope furnished no indication of disease of the lungs. After having suffered as above described, with little variation, for three years and a half, the patient dies from suffocation, some particles of bread and milk which he had taken for breakfast having fallen into the larynx.

On dissection, the trachea, just below the cricoid cartilage, was found contracted to the size of a goose-quill, the contraction being quite independent of adventitious deposit of any kind, the product of inflammation. The tracheal rings had entirely disappeared from the strictured part, whilst below, the constrictions of the rings were somewhat dilated beyond their natural calibre.

The alæ of the thyroid cartilage were somewhat approximated.

The author considers it probable that the disease had a syphilitic origin, and that the contraction of the membranous part of the trachea was consequent upon the absorption of the cartilaginous rings, and the simple result of the want of antagonism from the latter.—*Provin. Med. and Surg. Journ.*, Jan. 29, 1842.

---

*Amputation at the Hip-joint.*—At a meeting of the Academy of Sciences, Paris, January 3d, M. Larrey presented a report on a case of amputation at the hip-joint performed by M. Sedillot; this is the first successful operation of this kind ever performed at Paris.

The patient was a soldier, 28 years of age, who had compound fracture of the right thigh, in July, 1837. After having suffered for a considerable time from the results of the accident, union took place, but the injured limb was again accidentally broken. The patient was sent from the country to Paris in the early part of 1840. The leg was shortened, atrophied, and completely immovable; the thigh covered with cicatrices and several fistulous sores; the femur was carious in several places, and the disease could be traced communicating with the hip-joint. The limb was removed on the 7th August, by the double flap operation. The patient was discharged well in 50 days, and now enjoys excellent health.

M. Sedillot asks whether this operation should be performed at an early period, and before the developement of secondary accidents, or whether it is not more prudent to wait as he did, until the secondary effects of the injury have entirely disappeared. The author adopts the latter mode of practice. M. Larrey, on the other hand, affirms that primary amputation at the hip-joint may be performed in cases of recent injury as well as in those of chronic disease, and attributes the want of success in the former cases to the severe nature of the injury, on account of which the operation is usually required. M. Larrey also mentioned that he had performed this operation in two cases; in both, the wounds had been completely healed, when the unfortunate patients perished from cold and deprivations of every kind.—*Ibid.*

